# Seropositivity is associated with insulin resistance in patients with early inflammatory polyarthritis: results from the Norfolk Arthritis Register (NOAR): an observational study

**DOI:** 10.1186/ar3476

**Published:** 2011-09-29

**Authors:** Hoda Mirjafari, Tracey M Farragher, Suzanne MM Verstappen, Allen Yates, Diane Bunn, Tarnya Marshall, Mark Lunt, Deborah PM Symmons, Ian N Bruce

**Affiliations:** 1 Arthritis Research UK Epidemiology Unit, University of Manchester, Manchester Academic Health Sciences Centre, Oxford Road, Manchester, M13 9PT, UK; 2 Department of Biostatistics, University of Liverpool, Brownlow Street, Liverpool, L69 3GS, UK; 3 Clinical Research Department, Manchester Royal Infirmary, Oxford Road, Manchester, M13 9WL, UK; 4 Norfolk Arthritis Register, Norfolk and Norwich University Hospital, Colney Lane, Norwich, NR2 3SR, UK

## Abstract

**Introduction:**

Cardiovascular disease (CVD) is the leading cause of death in patients with inflammatory polyarthritis (IP), especially in seropositive disease. In established rheumatoid arthritis (RA), insulin resistance (IR) is increased and associated with CVD. We investigated factors associated with IR in an inception cohort of patients with early IP.

**Methods:**

Patients with early IP (two or more swollen joints for four or more weeks), aged 18 to 65 years, seen within 24 months of symptom onset were recruited from the Norfolk Arthritis Register (NOAR), a primary-care-based inception cohort. Assessment included joint examination, current and prior therapy and completion of the Health Assessment Questionnaire. Fasting blood was taken for measurement of CVD risk factors, rheumatoid factor (RF), anti-citrullinated protein antibodies (ACPA), C-reactive protein (CRP), and insulin levels. IR was calculated using the homeostatic model assessment (HOMA-IR). We examined factors associated with IR using univariate and multivariable linear regression models.

**Results:**

A total of 196 patients, including 59 (30%) males, were studied with a median (interquartile range, IQR) age and IP symptom duration of 49 (40 to 57) years and 6.7 (4.6 to 10.7) months, respectively. After age and gender adjustment, HOMA-IR was associated with obesity, (β-Coefficient (95% CI); 1.60 (0.96, 2.24)), higher systolic and diastolic blood pressure (0.03 (0.01, 0.05) and 0.04 (0.01, 0.08) respectively), triglycerides (1.06 (0.54, 1.57)), and HDL (-1.38 (-2.17,-0.58)). HOMA-IR was associated with serological status and this association persisted after adjustment for classic CVD risk factors and other IP-related variables (RF β-Coefficient (95% CI); 0.87 (0.20, 1.53) and ACPA β-Coefficient (95% CI); 1.42 (0.70, 2.15)).

**Conclusions:**

Seropositivity for RF or ACPA was associated with IR in this early IP cohort. This association may, in part, explain why seropositive patients have excess CVD mortality.

## Introduction

Cardiovascular disease (CVD) remains the leading cause of death in patients with inflammatory polyarthritis (IP) and is particularly associated with seropositive disease [1-4]. Insulin resistance (IR) is known to be increased in patients with established RA [5,6] and has been shown to be a risk factor for both clinical CVD [7] and subclinical atherosclerosis [8-10]. It remains unclear, however, whether IR occurs early in the course of IP or whether it develops later in the disease as a consequence of drug therapy, especially steroid exposure, physical inactivity or changes in body habitus, such as increased body fat:muscle ratio.

Established risk factors for IP development include smoking and obesity, both of which are also risk factors for CVD and have been associated with IR in the general population. It is, therefore, reasonable to consider whether IR relates primarily to these factors rather than to the inflammatory disease process *per se *[11]. There is also evidence that the association of IR with established RA may be mediated through the effects of systemic inflammation and/or glucocorticoid therapy [12]. Reduction in inflammatory biomarkers via glucocorticoid therapy, disease modifying anti-rheumatic drugs (DMARDs), anti-tumour necrosis factor (TNF)-α therapy or weight loss have all been associated with improvement in IR in RA [13-17]. It is, therefore, difficult to fully determine the direction of any associations found in established RA and what the key factor(s) related to IR are in this population.

The aim of this study, therefore, was to investigate the prevalence of IR in patients with early IP and to determine whether IR was associated with IP-related factors. In particular we were interested in examining if IR was related to inflammatory disease burden, serological status or early therapy exposure.

## Materials and methods

### Setting

The Norfolk Arthritis Register (NOAR) recruits individuals aged 16 years or older at symptom onset, who have swelling of at least two joints persisting for at least four weeks. Patients are notified to NOAR by primary care physicians or hospital rheumatologists in the catchment area [18]. A subset of consecutive patients recruited between January 2004 and December 2008 by the main NOAR cohort were also enrolled into this CVD sub-study if they were 18 to 65 years old and assessed within 24 months of joint symptom onset. Informed consent was obtained from patients and Norfolk Research Ethics Committees approval.

### Manifestations of inflammatory polyarthritis

At inclusion into the NOAR cohort, patients were interviewed by a research nurse. Current and previous medications for IP, as well as start and stop dates, were established. Patients were considered to have been exposed to a therapy if they reported any current or prior use. Fifty-one joints were assessed for the presence of swelling and tenderness. Fasting blood was collected, separated and frozen at -80°C in Norfolk before being transported to the Arthritis Research UK Epidemiology Unit in Manchester, UK, for further analysis. A Hitachi (BMG Labtech LTD, Aylesbury, UK) 917/911 automated analyser was used to determine C-reactive protein (CRP) concentration. The rheumatoid factor (RF) was measured using a particle enhanced immunoturbidimetric assay where > 40 iU/ml was considered positive for RF (Orion Diagnostica, BMG Labtech LTD, Aylesbury, UK). Antibodies to citrullinated protein antigens (ACPA) were measured using the Axis-Shield DIASTAT kit (Axis-Shield, Dundee, UK) where > 5 U/ml was considered positive for ACPA. The 28-joint Disease Activity Score (DAS28) was calculated based on 28 tender joint count, 28 swollen joint count, CRP and visual analogue scale (VAS) for general well-being [19]. The UK version of the Health Assessment Questionnaire (HAQ) was completed by the patient [20]. The 1987 American College of Rheumatology (ACR) classification criteria for RA were applied [21].

### Cardiovascular risk factors

Patients were classified as never smokers, previous smokers (if they had stopped smoking prior to the interview) or current smokers. Measurement of height and weight was carried out to calculate body mass index (BMI). Individuals were classified as being obese if their BMI was ≥ 30 kg/m^2^. Diabetes was considered to be present if patients reported a physician diagnosis of diabetes, if they were on treatment for diabetes, or if their fasting blood glucose was ≥ 7.1 mmol/L on the day of assessment. Total cholesterol, high density lipoprotein (HDL) and triglycerides were assayed on fresh fasting serum using CHOD-PAP, a homogenous direct method (Abbott Diagnostics, Berkshire, UK) and GPO-PAP methods respectively in Norfolk. LDL levels were mathematically derived from the total cholesterol and HDL values.

### Insulin resistance

Serum insulin levels were analysed using an ELISA kit from DRG diagnostics (Immunodiagnostic Services, Boldon, UK) on fasting frozen serum samples in Manchester. Serum insulin was measured by sensitive ELISA (Immunodiagnostic Systems Ltd, Boldon, UK). The kit employs a monoclonal antibody to human insulin, which shows no cross-reactivity to proinsulin. Insulin standards were calibrated against the World Health Organisation (WHO) international reference preparation 66/304. The analytical sensitivity of the assay was 1.76 mIU/L and intra and inter assay coefficients of variation were < 3.0% and < 6.0% respectively. The manufacturer's reference range in apparently normal people is 2 to 25 mIU/L. Insulin resistance was calculated using the Homeostasis Model Assessment, a model which allows derivation of insulin resistance (HOMA-IR) and pancreatic beta cell function (HOMA-B), calculated from fasting insulin/glucose pairs using homeostasis model assessment software, HOMA2, downloaded from the Diabetes Trials Unit, University of Oxford. This is an algorithm modified from the original by Jonathan Levy (Ref. Levy JC, Matthews DR and Hermans MP. Correct Homeostasis Model Assessment (HOMA) Evaluation uses the computer program *Diabetes Care *1998, **21:**2191-2192) IR was defined using the homeostatic model assessment (HOMA-IR): (Fasting insulin μU × Fasting glucose mmol/ml)/22.5.

Patients with a HOMA-IR value of ≥ 2.29 were classified as having IR as recommended in the literature [22].

### Statistical analysis

The baseline characteristics of patients with normal IR levels and patients with high IR levels were compared. For continuous variables, histograms were examined to ascertain if the variables were normally distributed. A *t*-test and Mann-Whitney U tests were used accordingly to compare demographic and clinical characteristics. Categorical variables were compared using the χ^2 ^test. Linear and logistic regression analyses, with adjustment for age and gender, were used to assess the association between the various traditional risk factors (TRFs) for CVD and IP related parameters with HOMA-IR and IR respectively. Linear and logistic regression was used to assess the association between RF and HOMA-IR and IR status as a binary variable, respectively. This was repeated for ACPA. Four groups were identified according to their serological status, that is: 1) negative for both RF and ACPA, 2) RF positive only, 3) ACPA positive only and 4) positive for both RF and ACPA. Linear regression using these four categories as the independent variable was used to examine the association between autoantibody status stratified into the above four categories and IR. All analyses were repeated, adjusting for the presence of TRFs for CVD and IP related parameters. The log likelihood test was used to analyse the degree of difference between the four groups and their association with IR. All analyses were carried out using the Stata 10 software package (Stata, College Station, TX, USA).

## Results

### Clinical characteristics of the cohort

We studied 196 patients, including 59 (30%) males, with a median (IQR) age and IP symptom duration of 49 (40 to 57) years and 6.7 (4.6 to 10.7) months respectively. Baseline characteristics are summarised in Table 1. Of note, 90 (47%) were RF positive, 66 (34%) were ACPA positive and 87 (44%) fulfilled 1987 ACR criteria for RA at baseline. The median (IQR) HOMA-IR was 2.7 (1.8 to 3.9) in the entire cohort and 118 (60%) were insulin resistant (HOMA-IR ≥ 2.29). Patients with IR had a higher prevalence of obesity, higher blood pressure and triglyceride levels and lower HDL levels. A higher proportion of insulin resistant patients were RF or ACPA positive (Table 1).

**Table 1:** Characteristics of 196 patients with recent onset IP and in those with and without IR

Variable at baseline	All pts *n *= 196	IR (HOMA IR ≥ 2.29) *n *= 118 (60%)	No IR HOMA IR < 2.29 *n *= 78 (40%)	*P-*value
Age when first seen (years) ^†^	49 (40 to 57)	49 (41 to 56)	48 (37 to 57)	0.49
Male gender	59 (30%)	38 (32%)	21 (27%)	0.43
Current smoker	50 (26%)	27 (23%)	23 (29%)	0.30
Obese (BMI ≥ 30)	57 (30%)	46 (40%)	11 (15%)	< 0.001
On treatment for or diagnosed with DM	26 (13%)	16 (14%)	10 (13%)	0.88
Fasting blood glucose (mmol/L) *	4.7 (4.4 to 5.0)	4.8 (4.5 to 5.1)	4.5 (4.3 to 4.8)	< 0.001
Fasting insulin (μU)	12.7 (8.9 to 18.1)	16.7 (13.2 to 21.6)	8.2 (6.0 to 9.4)	< 0.001
HOMA-IR	2.7 (1.8 to 3.9)	3.5 (2.8 to 4.8)	1.6 (1.2 to 1.9)	< 0.001
SBP (mmHg) *	132 (17)	135 (17)	129 (15)	0.04
DBP (mmHg) *	81 (10)	83 (10)	79 (9)	0.01
On treatment for hypertension	19 (10%)	13 (11%)	6 (8%)	0.44
TG (mmol/L) *	1.4 (0.6)	1.6 (0.6)	1.1 (0.5)	< 0.001
T.Chol (mmol/L) *	5.4 (1.1)	5.5 (1.1)	5.2 (1.1)	0.24
HDL (mmol/L) *	1.5 (0.4)	1.5 (0.5)	1.6 (0.4)	0.03
LDL (mmol/L) *	3.2 (0.9)	3.3 (0.9)	3.1 (0.9)	0.43
On statin treatment	11 (6%)	5 (4%)	6 (8%)	0.30
IP symptom duration (months)^†^	6.7 (4.6 to 10.7)	6.5 (4.5 to 9.9)	7.0 (4.7 to 11.8)	0.28
Age at IP symptom onset	48 (40 to 56)	48 (40 to 55)	48 (36 to 57)	0.42
RF positive	90 (47%)	63 (55%)	27 (35%)	0.01
ACPA positive	66 (34%)	50 (44%)	16 (21%)	< 0.001
Fulfil ACR criteria for RA	87 (44%)	58 (49%)	29 (37%)	0.10
Swollen joint count (/51)^†^	4 (1 to 7)	4 (2 to 9)	4 (1 to 7)	0.44
Tender joint count (/51)^†^	8 (2 to 18)	8 (2 to 18)	9 (3 to 17)	0.87
Both swollen & tender (/51)^†^	2 (0 to 6)	2 (0 to 6)	2 (0 to 5)	0.96
CRP (mg/L)^†^	10 (7 to 17)	11 (5 to 21)	9 (7 to 13)	0.21
DAS28_CRP _^†^	3.9 (3.0 to 4.8)	3.9 (3.0 to 4.9)	3.9 (3.1 to 4.7)	0.73
HAQ^†^	0.88 (0.38 to 1.38)	0.88 (0.38 to 1.68)	0.88 (0.38 to 1.38)	0.26
On steroids prior to baseline assessment	43 (22%)	24 (20%)	19 (24%)	0.51
On DMARDs prior to baseline assessment	105 (54%)	67 (57%)	38 (49%)	0.27
On MTX prior to baseline assessment	60 (31%)	36 (31%)	24 (31%)	0.97
On HCQ prior to baseline assessment	11 (6%)	6 (5%)	5 (6%)	0.69

All data are presented as n (%) except where we indicate that either median (IQR) ^† ^or mean (SD) * were used.

ACPA, anti-CCP antibody; ACR, American College of Rheumatology; CRP, C-reactive protein; DAS 28_CRP _, Disease Activity Score calculated using CRP; DBP, diastolic blood pressure; DM, diabetes mellitus; HAQ, Health Assessment Questionnaire; HCQ, hydroxychloroquine; HDL, high density lipoprotein; HOMA-IR, Homeostatic Model Assessment for Insulin Resistance; IP, inflammatory polyarthritis; LDL, low density lipoprotein; MTX, methotrexate; RF, rheumatoid factor; SBP, systolic blood pressure;

T.chol, total cholesterol; TG, triglycerides

### Factors associated with insulin resistance

In an age and gender adjusted linear regression analysis, HOMA-IR was significantly associated with a number of established cardiovascular and metabolic factors including obesity, systolic and diastolic blood pressure, triglyceride levels and HDL. These associations were also seen when we considered IR as a dichotomous outcome (Table 2).

**Table 2:** Associations between traditional cardiovascular disease risk factors, IP related factors and insulin resistance after adjustment for age and gender

*Variable*	*Dependent variable ^†^*	
	HOMA-IR (continuos) *^▲^* *(β-Coefficient (95% CI))*	IR* (HOMA-IR > 2.29)*^#^* *(Odds ratio (95% CI))*
Age at time of study (per year)	0.018 (-0.012, 0.048)	1.02 (0.99, 1.04)
Male gender	0.044 (-0.665, 0.753)	1.27 (0.67, 2.39)
Current smoker	0.004 (-0.747, 0.755)	0.71 (0.37, 1.37)
Obese (Y/N)	**1.602 (0.963, 2.240)**	**4.02 (1.91, 8.50)**
SBP (per mmHg)	**0.029 (0.007, 0.050)**	**1.02 (1.00, 1.04)**
DBP (per mmHg)	**0.043 (0.009, 0.077)**	**1.04 (1.01, 1.08)**
TG (per mmol/L)	**1.057 (0.542, 1.572)**	**3.95 (2.09, 7.46)**
T.Chol (per mmol/L)	0.155 (-0.162, 0.472)	1.19 (0.90, 1.59)
HDL (per mmol/L)	**-1.375 (-2.172, -0.578)**	**0.44 (0.21, 0.92)**
LDL (per mmol/L)	0.193 (-0.169, 0.556)	1.11 (0.80, 1.53)
DM (On treatment for DM/DM/fasting glucose ≥ 7.1 mmol/L)	-0.114 (-1.170, 0.942)	0.73 (0.29, 1.82)
IP disease duration (per month)	-0.001 (-0.003, 0.001)	1.00 (1.00, 1.00)
RF positive	**0.924 (0.254, 1.594)**	**2.20 (1.20, 4.06)**
ACPA positive	**1.051 (0.336, 1.767)**	**3.00 (1.51, 5.96)**
ACR criteria for RA	0.542 (-0.114, 1.198)	1.62 (0.90, 2.93)
Swollen joint (per joint)	0.032 (-0.016, 0.080)	1.03 (0.98, 1.07)
Tender joint (per joint)	**0.029 (0.002, 0.056)**	1.01 (0.99, 1.04)
CRP (per mg/L)	0.007 (-0.012, 0.026)	1.00 (0.99, 1.02)
DAS28	0.168 (-0.100, 0.437)	1.05 (0.83, 1.34)
HAQ	**0.709 (0.237, 1.182)**	1.41 (0.91, 2.19)
On steroids prior to baseline assessment	-0.300 (-1.094, 0.493)	0.74 (0.37, 1.48)
On DMARDs prior to baseline assessment	0.533 (-0.124, 1.190)	1.31 (0.73, 2.34)
On MTX prior to baseline assessment	0.277 (-0.435, 0.989)	0.94 (0.50, 1.76)
On HQC prior to baseline assessment	-0.218 (-1.636, 1.200)	0.81 (0.24, 2.79)

^▲^Linear regression producing β-coefficients were used for continuous outcome. β-coefficients are considered statistically significant if their 95% CI values do not include zero. **^#^**Logistic regression producing odds ratios used for binary outcomes. Odds ratios are considered significant if the 95% CI values do not include 1. † (adjusted for age and gender)

ACPA, anti-CCP antibody; ACR, American College of Rheumatology; BMI, body mass index; CRP, C-reactive protein; DAS, Disease Activity Score; DBP, diastolic blood pressure; DM, diabetes mellitus; DMARDs, disease modifying anti-rheumatic drugs; HAQ, Health Assessment Questionnaire; HCQ, hydroxychloroquine; HDL, high density lipoprotein; IP, inflammatory polyarthritis; LDL, low density lipoprotein; MTX, methotrexate; RF, rheumatoid factor; SBP, systolic blood pressure; T.Chol, total cholesterol; TG, triglycerides.

HOMA-IR was associated with tender joint counts, and HAQ score (β-Coefficient (95% CI); 0.029 (0.002, 0.056) and 0.709 (0.237, 1.182), respectively). In addition, patients who were seropositive for RF or ACPA had a significantly higher HOMA-IR score (β-Coefficient (95% CI); 0.924 (0.254, 1.594) and 1.051 (0.336, 1.767) respectively) and were more likely to be insulin resistant (Table 2). The association between RF and ACPA status and HOMA-IR remained after adjustment and/or removal of patients with known diabetes mellitus, those already taking DMARD or steroid therapy, and after adjustment for other CVD risk factors and IP-related factors examined (fully adjusted β-Coefficient (95% CI) for RF = 0.867 (0.204, 1.530) and ACPA = 1.423 (0.701, 2.146) respectively).

Both RF and ACPA status were associated with HOMA-IR. In the small number of patients positive for only RF or ACPA (15% and 3% respectively) there was no association with HOMA-IR. However, there was a significant association between being positive for both RF and ACPA and HOMA-IR (Table 3). Patients with both RF and ACPA had a significantly stronger association with HOMA-IR than the RF positive group (log likelihood test (*P *= 0.0061) (Table 3).

**Table 3:** Associations between rheumatoid factor and anti-CCP antibodies with insulin resistance alone and in combination with one another after serial adjustment

*Variable *	*Numbers of patients*	*Association with HOMA-IR* *TRF & IP risk factor adjusted IR* *β-Coefficient OR (95% CI) ^#^*
RF +ve *	90 (47%)	**0.867 (0.204, 1.530)**
ACPA +ve *	66 (35%)	**1.423 (0.701, 2.146)**
RF-ve/ACPA -ve	96 (49%)	0
RF +ve/ ACPA -ve ^†^	29 (15%)	-0.015 (-0.973, 0.944)
ACPA+ve/ RF -ve^†^	6 (3%)	0.932 (-0.898, 2.763)
Both RF+ve/ACPA+ve ^†^	60 (31%)	**1.472 (0.695, 2.250) **^√^

* = Seropositive *vs*. seronegative for RF and ACPA (*n *= 193 and 191, respectively)

† = Patients stratified into four groups depending on autoantibody status and compared with other groups (*n *= 191)

√ = Model significantly different in patients positive for both RF and ACPA relative to those RF positive only (*P *= 0.0061) with age and gender and other parameters adjusted.)

^▲^Linear regression producing β-coefficients were used for continuous outcome. β-coefficients are considered statistically significant if their 95% CI values do not include zero.

***^#^***Logistic regression producing odds ratios used for binary outcomes. Odds ratios are considered significant if the 95% CI values do not include 1.

ACPA, anti-CCP antibody; IP, inflammatory polyarthritis; RF, rheumatoid factor.

One hundred (51%) patients were seropositive for RF or ACPA. Examination of this subset revealed similar associations with IR as was observed in the whole cohort including a significant association between HOMA-IR and tender joint counts and HAQ score (β-Coefficient (95% CI); 0.049 (0.004, 0.094) and 0.780 (0.071, 1.489), respectively) but not DAS28 score, CRP or swollen joint counts.

## Discussion

In this cohort of patients with IP we have found a significant association between serological status (RF and ACPA) and insulin resistance measured as HOMA-IR. This association persists after adjustment for classic cardiovascular risk factors and other IP-related factors. As far as we are aware, this is the first time this observation has been noted in an early IP population.

A number of previous studies have examined IR in the context of established RA, usually drawn from hospital cohorts. These studies have demonstrated increased IR in RA [7,23]. Most studies have demonstrated that insulin levels are associated with other metabolic factors generally clustered within the Metabolic Syndrome [9,24]. The association between therapy (steroids in particular) and insulin levels has, however, been controversial [23,25,26]. There is, however, evidence that IR may be related to inflammatory disease burden [27]. This association has been supported by observations of reduced insulin resistance following control of disease with anti-TNF therapy [15,16]. With regard to clinical outcomes, insulin resistance in RA has been associated with both subclinical atherosclerosis and clinical cardiovascular disease [7,28,29]. Given that most study series of RA patients will have a very high percentage who are seropositive, it may not be surprising that the association we have described in our study has not been observed in these contexts. In addition, several previous studies did not report ACPA status. Our previous studies have demonstrated that seropositivity is an important prognostic factor in patients with IP and we have shown that it is in this subgroup that there is a particular excess of CVD mortality [1,2,4].

In our cohort, IR was associated with the typical pattern of metabolic changes expected from other population studies. Higher levels of IR are associated with obesity, fasting blood glucose, blood pressure, triglycerides and low HDL. We also found a clear and consistent association between seropositivity for RF and ACPA with IR, whether this was measured across the range of HOMA-IR or if we treated IR as a categorical outcome. With regard to other IP-related parameters, we did find that higher levels of HOMA-IR were associated with tender joint counts and HAQ score at baseline; however, the lack of association with DAS28 scores, swollen joint counts and acute phase reactants argues against a strong influence of inflammatory disease burden in this cohort. Previous general population studies have also shown that TNF-α and IL-6 are increased in insulin resistant states [30,31]. TNF-α is known to interfere with both glycaemic sensing and insulin signalling, thus impairing glucose handling [31]. The observation by others that TNF blockade reduces IR, would also support the hypothesis that inflammatory disease burden contributes to IR in IP [32]. Our data, however, suggest that serological status may be a dominant factor determining levels of IR in inflammatory polyarthritis patients.

While severe insulin resistance secondary to insulin receptor antibodies in the context of type I diabetes mellitus is widely recognized [33], there are limited published data describing the association of insulin resistance with other autoantibodies. RA and its related conditions overlap with other autoimmune conditions and affect many organs. It is possible that the same triggers to autoimmune RF and ACPA production also contribute to insulin receptor antibodies sufficient to induce the degree of insulin resistance that we see in our study. We note a higher level of insulin production and, therefore, islet cell destruction is unlikely to be the mechanism by which this insulin resistance is induced.

We did not find any significant contribution of therapy to IR in our cohort. However, in this early cohort only 22% of patients were exposed to steroids by the time of the study and 54% of patients had been recently started on DMARD therapy. DMARDs may of course reduce IR through anti-inflammatory effects, and this may also even be true of low-dose steroid therapy [34]. We plan to follow this cohort to determine whether therapy does play a role in influencing the metabolic status of these patients over time.

Most of the association between serological status and IR remains unexplained in our fully adjusted models. We also note that the association was particularly strong in those who are positive for both antibodies, although it should be pointed out that this was the majority of seropositive patients. RF and ACPA are associated with the presence of the shared epitope, and can be positive for many years prior to the onset of arthritis. From a prognostic viewpoint, seropositivity also predicts future cardiovascular mortality risk [35,36]. Seropositive patients are also more likely to develop extra-articular features such as nodules, lung disease and vasculitis [37-39]. Other groups have found evidence for endothelial cell activation and dysfunction in seropositive patients [40,41] and in RA patients who carry the shared epitope [42]. Insulin resistance, therefore, may be a consequence of early endothelial dysfunction in seropositive patients. Insulin stimulates disposal of glucose from the circulation into skeletal muscle and in eNOS knockout mice, capillary density is reduced and insulin mediated glucose clearance is reduced by 40% [43]. Therefore, endothelial dysfunction is hypothesised to be a primary step in the development of the insulin resistant state.

The development of RF and ACPA has also been associated with the shared epitope, and with smoking status [1,44,45]. In addition, we have previously found that obesity is a risk factor for developing IP [46]. An alternative explanation for the observations found is that smoking and obesity contribute to both the IR and seropositivity in the population at risk of developing IP. Although our study looked at patients early in the course of IP, ultimately this question can only be answered in a pre-symptomatic population as we cannot accurately determine which came first in this population.

There are several limitations which should be considered in this study. First, our study is limited by its cross-sectional nature and longitudinal studies are required to examine in closer detail the temporal and causal relationship between adiposity, classic risk factors for CVD, inflammatory joint disease and therapy on the development of IR. Conversely, by examining this question in a community-based, early IP population, we have noted an important association between serological status and IR. Secondly, the limited number of patients did not allow for further stratification of our results, in particular with regard to the dose and cumulative exposure of oral steroid therapy and its associations with IR. A further limitation is that we did not have the facilities in a community-based study to undertake hyperinsulinaemic euglycaemic clamp experiments, which are the 'gold standard' for ascertaining IR levels [22]. The HOMA-IR has, however, been validated as being an appropriate alternative from a blood sample collected from our patients in their own home [47]. Multiple testing using both HOMA-IR as a continuous variable and IR as a binary variable may be an issue. Whilst HOMA-IR may be considered more statistically valid, the use of IR as a binary variable is more clinically useful and so both outcomes are presented here, with results that were consistent using both approaches.

## Conclusions

In conclusion, we have found that in an early IP population, IR is associated with the presence of RF and ACPA, an effect that persists after adjustment for metabolic and other IP-related factors. Insulin resistance is, therefore, detectable early in the course of IP and may, in part, explain the excess CVD risk observed in seropositive patients. A better understanding of the mechanisms underlying this observation may shed light on the pathogenesis of accelerated atherosclerosis in IP patients.

## Abbreviations

ACPA: anticitrullinated protein antibodies; ACR: american college of rheumatology; BMI: body mass index; CI: confidence interval; CRP: c-reactive protein; CVD: cardiovascular; DAS28: disease activity score; DMARD: disease modifying anti-rheumatic drung; ELISA: enzyme linked immunosorbant assay; HAQ: health assessment questionnaire; HDL: high density lipoprotein; HOMA-IR: homeostatic model assessment of insulin resistance; IP: inflammatory polyarthritis; IQR: interquartile range; NOAR: norfolk arthritis register; RA: rheumatoid arthritis; RF: rheumatoid factor; TNF: tumour necrosis factor; TRFs: traditional risk factors; VAS: visual analogue scale.

## Competing interests

The authors declare that they have no competing interests.

## Authors' contributions

HM contributed to the design, data collection and analysis for this paper and wrote the manuscript. TF is the previous project lead and was involved in coordinating and supervising data collection, analysis and manuscript writing. SV is the project lead and coordinated and supervised data collection, analysis and manuscript writing. AY was the laboratory lead in analysing the serum insulin levels. DB is the Clinical Manager in charge of patient recruitment, consent, follow-up and data collection. TM is a Consultant Rheumatologist and is involved in patient recruitment for the study. ML is a Senior Lecturer in Statistics and contributed to study design and analysis. DS is the founder and principal investigator on the NOAR study. IB is the co-principal investigator and was instrumentally involved in study design, analysis and manuscript preparation. All authors have read and approved the manuscript for publication.

## Authors' information

HM is a Clinical Research Fellow at the Arthritis Research UK Epidemiology Unit at the University of Manchester.
